# Economy

**Published:** 1875-12

**Authors:** 


Economy. — As a rule of human conduct, economy is a virtue that should be cultivated. But these are exceptional times. The difficulty now is not that money is scarce—for the banks are full of it—but that it does not circulate. In flush times display by the rich is wrong, because the example thus set is certain to be followed by those of smaller means, and the appetite once whetted soon outgrows the means to supply it. But the theories of universal economy do not apply, when, as now, circumstances change. The question now is not whether the diminished purse can afford its possessor this or that luxury, but whether it will furnish what is actually necessary; and the rich, instead of adding to wealth —already more than adequate to their greatest wants—by accumulation, should scatter their money abroad, stimulate industry, and set the wheels of business whirling with fresh vigor. When the poor are so poor that they cannot economize, and the rich must either feed them in idleness or provide work for them to earn food, which shall they do?

It is not charity but work which the honest man now needs. It is not his *example* that the small tradesman and manufacturer and the laborer for daily bread are in want of, but it is the use of the rich man's money, which, once loosened from its unproductive resting-place, will pass around the circle of waiting hands, and, with its increase, return to him precious with the blessings it has bestowed. This is true charity, and charity of the most profitable sort, all around. If every person in possession of more than enough property to pay for three months' bread and shelter would adopt this system of charity to the extent of employing just one tenth of all he has, in building and other productive enterprises, the army of hungry mouths would be filled, and our land would smile with abundant prosperity.

